# Relationship Between Glycemic Indices and eGFR Values Among Type 2 Diabetes Mellitus Individuals With Chronic Kidney Disease Across Various Progression Stages

**DOI:** 10.1177/11795514251362516

**Published:** 2025-11-01

**Authors:** K. Vaishali, Chandana Acharya, Shobha U. Kamath, Revati Amin, Shivashankara Kaniyoor Nagri

**Affiliations:** 1 Department of Physiotherapy, Manipal College of Health Professions, Manipal Academy of Higher Education, Manipal, Karnataka, India; 2 Department of General Medicine, Kasturba Medical College, Manipal Academy of Higher Education, Manipal, Karnataka, India; 3 Department of Biochemistry, Kasturba Medical College, Manipal Academy of Higher Education, Manipal, Karnataka, India; 4 Department of Physiotherapy, Kasturba Medical College Mangalore, Manipal Academy of Higher Education, Manipal, Karnataka, India

**Keywords:** CKD, correlation, eGFR, fructosamine, glycated hemoglobin, HbA1c, T2DM

## Abstract

**Background:**

T2DM is a significant contributor to hypoglycemia, mortality, CVD, as well as a leading cause of CKD. Understanding and managing glycosylation discrepancies in T2DM patients with CKD is critical because they are important in disease causation and progression. Aim of this correlation study was to investigate the accuracy of HbA1c and fructosamine as predictors of declining renal function in the context of T2DM, by comparing and evaluating their relationships with eGFR across stages 1 to 5.

**Methods:**

We included individuals with T2DM aged over 18 years, diagnosed per ADA guidelines, and potential CKD (stage 1-5) in T2DM patients. Outcomes involved measuring HbA1c and fructosamine levels of all participants.

**Results:**

We recruited 424 participants from Department of Medicine, Kasturba Medical College, MAHE, Manipal on OPD & IPD. For patient in CKD stage 1 to 4, a weak positive correlation was noticed between HbA1c and eGFR. For patient in CKD stage 1 to 4, a weak negative correlation was found between eGFR and Fructosamine.

**Conclusion:**

For determining long-term blood sugar management and forecasting the course of kidney disease in individuals with type 2 diabetes, the HbA1c test remains a crucial and dependable tool. However, it might occasionally be more difficult to interpret HbA1c values in later stages of CKD. Fructosamine, a shorter-term blood sugar indicator, can offer useful further information in certain situations. We advise combining the 2 tests for optimal diabetes management: fructosamine for prompt medication modifications, particularly in advanced CKD and HbA1c for long term trends and risk assessments.

## Plain language summary

Relationship between glycemic indices and eGFR values among T2DM individuals with CKD

**Background:** Type 2 diabetes (T2DM) can lead to serious complications, including chronic kidney disease (CKD). Physicians often use blood tests like HbA1c (average blood sugar over 2 to 3 months) and fructosamine (average blood sugar over 2-3 weeks) to monitor diabetes control. However, in people with CKD, these tests may not work the same way. This study examines how well HbA1c and fructosamine reflect kidney function in different stages of CKD.

**Methods:** We studied 424 adults with Type 2 DM and CKD (stages 1-5). We measured their HbA1c and fructosamine and kidney function (eGFR) to see how these tests relate to each other across different stages.

**Results:** HbA1c showed a weak correlation with kidney function in early CKD (stages 1-4). Fructosamine had a slightly stronger correlation between kidney functions than HbA1c in these stages. In advanced CKD (stage 5), HbA1c may be less reliable, while fructosamine could still be useful. HbA1c It’s still important for long term diabetes monitoring but may need extra caution in the later-stage CKD. Fructosamine Can help when HbA1c is unclear, especially for short term treatment adjustments. Using both tests together may give a more complete picture of diabetes control in CKD patients.

**Conclusion:** For people with diabetes and kidney diseases, combining HbA1c and fructosamine can improve blood sugar monitoring and treatment decisions. This approach may help protect kidney health and prevent complications.

## Introduction

Type 2 diabetes mellitus (T2DM) is acknowledged as an important contributor of cardiovascular diseases (CVD), hypoglycemia, chronic kidney disease (CKD), and mortality on the global level.^1-3^ In patients who have both conditions, poor management of T2DM increases the probability of developing CKD, which in turn increases morbidity and mortality.^4-6^ The prevalence of T2DM-related CKD has increased in recent decades, emphasizing the crucial need for early detection, primary care therapies, and consistent follow-up, particularly in socioeconomically disadvantaged areas, to prevent serious adverse effects.^2,3,7^

Developed countries have greater global incidences of T2DM-related CKD, mortality rates, and Disability-Adjusted Life Years (DALYs).^8,9^ The prevalence of T2DM has risen globally, especially in countries that are members of the European Union.^10^ Since 1990, the prevalence of CKD caused by T2DM has grown, with greater rates found in regions with a middle socioeconomic index and industrialized countries, and lower rates in poorer and middle Human Development Index countries.^3^

In India, CKD is common among T2DM patients, with studies indicating 17.2% nationwide and 32.2% in Andhra Pradesh.^12^ As a primary cause of renal failure and an important predictor for CVD, T2DM doubles or quadruples the chance of CV complications and death.^13,14^ Individuals with T2DM are predisposed to serious complications, including nephropathy.^15^

T2DM and CKD are linked by complicated biological pathways. Studies show that T2DM patients with relatively small glomerular filtration rate (GFR) impairment had greater atherogenic lipid profiles than those with normal renal function, implying that lipid abnormalities play a role in the T2DM-CKD relationship.^16^

To prevent vascular complications, T2DM patients must closely monitor crucial quality indicators like glycated hemoglobin (HbA1c), systolic blood pressure (SBP) and low-density lipoprotein cholesterol (LDL-C).^17^ However, establishing optimal management remains difficult. Research indicates that only 51.8% of prediabetic individuals have moderate to high cardiovascular disease (CVD) risk scores, predisposing them to developing T2DM.^18^

Glycosylation, the enzymatic attachment of sugar molecules to proteins and lipids, is a common post-translational modification that influences biological processes.^19^ In type 2 diabetes, hyperglycemia causes non-enzymatic glycation, which increases protein glycosylation. This leads to the accumulation of Advanced Glycation End Products (AGEs) in tissues, which exacerbates the complications of diabetes, including CKD.^20^

Glycemic indices refer to biomarkers of glycemic control (HbA1c and fructosamine) rather than the glycemic index of foods. This distinction is important to avoid confusion with the dietary glycemic index, which measures the effect of carbohydrates on blood glucose levels. HbA1c and fructosamine are biomarkers due to their widespread use in clinical practice for monitoring glycemic control. While fructosamine is less reliable than HbA1c in predicting long-term glycemic control, it provides a shorter-term glycemic index 2-3 weeks) and can be useful in patients with conditions that affect HbA1c reliability, such as anemia or hemoglobinopathies.

In CKD, lower renal filtration capacity causes AGEs retention, which promotes inflammation, oxidative stress, and fibrosis, increasing kidney damage.^21^ Abnormal glycosylation can also affect the structure and function of important renal proteins, such as podocyte proteins in the glomerular filtration barrier, leading to increased permeability and proteinuria, which are significant indications of CKD development.^22^

Understanding and managing glycosylation discrepancies in T2DM patients with CKD is critical because they are important in disease causation and progression. However, there is little knowledge about glycosylation changes in T2DM patients at various CKD stages, indicating a crucial research gap. This study seeks to address this gap by examining glycosylation patterns at various stages of CKD in T2DM patients. The findings are expected to inform individualized treatment regimens and improve patient outcomes. The goal is to understand how these markers of glycemic control relate to each other across the different stages of CKD, considering the unique metabolic and physiological changes that occur with CKD progression. The purpose of the study was to determine the predictive power of HbA1c, fructosamine, and eGFR (estimated glomerular filtration rate) biomarkers for declining renal function in the context of T2DM by examining their associations across CKD stages 1 to 5. The study has 2 objectives. The first is to determine the relationship between HbA1c and eGFR in each stage of CKD stage 1 to 5 in T2DM. The second objective is to determine the relationship between fructosamine and eGFR in each stage of CKD stage 1 to 5 in T2DM individuals.

## Methods

This is a cross-sectional study. The study was conducted based on Strengthening the reports of observational studies in epidemiology (STROBE) statement: guidelines for reporting observational studies for reporting the findings of the study.^23^ Participants were diagnosed with diabetes following the American Diabetes Association (ADA) 2010 criteria,^24^ with comprehensive assessments. CKD was diagnosed and staged according to the KDIGO 2012 guidelines,^25^ incorporating criteria such as renal structure/ function abnormalities persisting for above 3 months and GFR and albuminuria categories.

Participants were categorized into stages according to their eGFR values (Table 1).

**Table 1.** Estimated Glomerular Filtration Rate (eGFR) Classification for CKD Stages.

eGFR value	CKD stage	Description
⩾90 mL/min	Stage 1	Normal
89-60	Stage 2	Mild CKD
59-30	Stage 3	Moderate CKD
29-15	Stage 4	Severe CKD
<15	Stage 5	End stage CKD

Abbreviations: CKD, chronic kidney disease; eGFR, estimated glomerular filtration rate.

With a cohort of 424 individuals the sample size was calculated using mean comparison from a previous study by Neelofar and Ahmad,^26^ using the formula n = (Zα/2 + Zβ)2 × 2 × σ2/d2. Participants were divided into 5 groups, each consisting of 106 individuals, categorized according to the stage of CKD in diabetes patients.

Inclusion criteria encompassed individuals with T2DM above 18 years of age, diagnosed per ADA guidelines, and potential CKD (stage 1-5) in T2DM patients. Exclusion criteria were detailed and excluded individuals with specific medical conditions or circumstances that could confound the study outcomes.

Laboratory analyses involved measuring glycated hemoglobin (HbA1c) and fructosamine levels using specific methods, along with comprehensive clinical assessments including fasting and postprandial blood glucose levels, serum creatinine, and albumin:creatinine ratio. CKD staging relied on estimated GFR derived from the CKD EPI creatinine equation 2009.

Laboratory Methods: HbA1c was measured using high-performance liquid chromatography (HPLC; Bio-Rad D-10™). Fructosamine was quantified via the nitrobluetetrazolium (NBT) colorimetric assay (Roche Diagnostics).

Blood Collection & Processing: Samples were collected after 8-hour fast HbA1c in EDTA tubes and fructosamine in serum tubes. All samples were processed within 2 hours to ensure stability.

CKD Stading & Recruitment: eGFR was calculated using the CKD-EPI creatinine equation (2009). Albuminuria was assessed per KDIGO 2012 guidelines (UACR ⩾ 30 mg/g). Early stage (1-2) CKD participants were identified through routine screening (eg, annual check-ups, diabetes/hypertension clinics) despite being asymptomatic, as per standard CKD surveillance protocols. Early-stage CKD patients (stages 1-2) were identified through routine diabetes care protocols, which include annual eGFR and urine albumin-to-creatinine ratio (UACR) screening per ADA guidelines, even in asymptomatic individuals.

Institutional Ethics Committee (IEC) at Kasturba Medical College and Kasturba Hospital (708/2018) granted permission to conduct this study, and it was registered prospectively in the Clinical Trial Registry of India (CTRI/2019/02/017566). All participants were received at the Department of Medicine, Kasturba Medical College, MAHE, Manipal, during outpatient (OPD) or inpatient (IPD) visits from February 2019 to February 2021. The participants were evaluated and recruited for the study based on their eligibility. Each participant was given a comprehensive explanation of the study before providing written consent to participate. Based on their baseline eGFR readings, all individuals were divided into Stages 1 to 5. After categorization, all participants underwent baseline assessment and data was obtained for the outcome variables that is, eGFR, HbA1c and fructosamine.

## Data Analysis

The data was analyzed using Jamovi software (version 2.2.5.). The Shapiro-Wilk test was used to determine whether the data was normal. The demographic characteristics for continuous data were presented as mean, median, standard deviation, and interquartile ranges (Q1 and Q4) based on the normality results, while frequencies and percentages were used to summarize categorical variables. The association between HbA1c, fructosamine and eGFR was investigated using Spearman’s rank correlation test for all 5 stages of CKD. The correlation’s strength was determined by analyzing the correlation coefficient’s (*r*) absolute value. A strong correlation was defined as *r* ⩾ .6, a moderate correlation as .3 ⩽ *r* < .6, and a weak correlation as *r* = < .3. Statistical significance was determined at *P* < .05.

Multivariate linear regression was performed to assess associations between glycemic indices (HbA1c, fructosamine) and eGFR, adjusting for age, diabetes duration, and anemia status. Results are reported as β-coefficients with 95% confidence intervals (CIs).

We accounted for multiple comparisons using the Bonferroni correction, adjusting the significance threshold (α) to .01 (.05/5) for our primary analysis across 5 CKD stages. Secondary analysis maintained α = .05 with explicit notation of unadjusted *P*-values.

## Results

**Figure 1.**
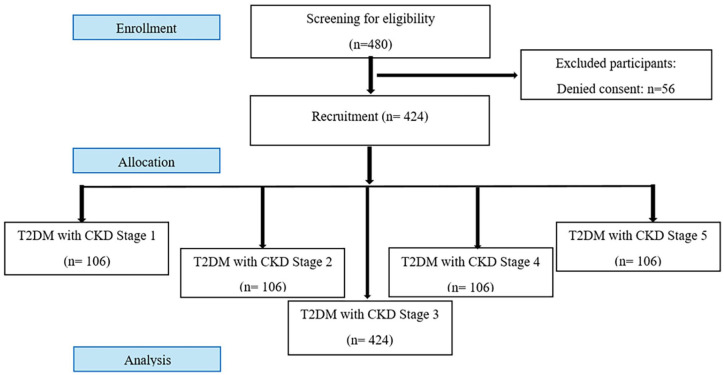
CONSORT flow diagram.

Participants in the study included 424 CKD individuals with T2DM. We categorized 424 participants each into 5 groups that is, CKD stage 1 to 5 (Figure 1). The demographic characteristics of all participants in each CKD stage is described in Table 2.

**Table 2.** Demographic Clinical Characteristics of T2DM Patients With CKD All Progression Stages (Stages 1-5).

Variable	CKD 1		CKD 2		CKD 3		CKD 4		CKD 5	
	Mean (SD)/Median (Q1-Q4) N (%)	*P* value	Mean (SD)/Median (Q1-Q4) N (%)	*P* value	Mean (SD)/Median (Q1-Q4) N (%)	*P* value	Mean (SD)/Median (Q1-Q4) N (%)	*P* value	Mean (SD)/Median (Q1-Q4) N (%)	*P* value
Age, years	57.41 (3.41)	<.001	63 (58-69)	.003	64 (3.37)	<.001	64.42 (4.1)	<.001	64.32 (3.6)	.002
Gender										
Male	98 (89.9%)		100 (91.7%)		93 (85.3%)		96 (88.07%)		99 (90.8%)	
Female	11 (10.0%)		9 (8.3%)		16 (14.67%)		13 (11.9%)		10 (9.17%)	
Weight, kg	65.33 (6.14)	<.001	58.7 (46.09)	.001	60.18 (13.3)	.002	67.32 (6.807)	<.001	69.2 (5.6)	.002
Height, cm	163(152.9-172.6)	.005	162.15(150.3)	.002	167.7 (7.17)	<.001	165.37 (7.28)	<.001	164.7 (152.8-178.3)	.012
BMI, kg/m^2^	24.5 (19-30.4)	<.001	22.3 (15.4-30.46)	.300	21.13 (15.55-28.81)	.247	24.3 (19.2-30.96)	.009	25.53 (20.28-33.42)	.128
Post-prandial plasma glucose, mg/dL	160.0 (99.68)	<.001	176.14 (143.7)	.003	179.01 (96.96)	<.001	180.22 (117.66)	<.001	179.93 (104.13)	.002
Fasting glucose, mg/dL	245.2 (240.6-250.2)	.506	239.3 (15.5)	.003	224.5 (0.98)	<.001	207.12 (9.09)	<.001	193.26(175.46-218.18)	.092
Creatinine, mg/dL	0.738 (0.08)	<.001	1.08 (0.09)	<.001	1.75 (0.36)	<.001	3.49 (0.518)	<.001	6.05 (1.506)	<.001
HbA1c, mmol/mol	7.4 (7.2-7.8)	.052	7.86 (0.15)	<.001	8.48 (0.02)	<.001	7.99 (0.25)	<.001	7.6 (7.1-8.3)	.092
Fructosamine, µmol/L	395.06 (382.19-406.3)	.807	318.03 (78.282)	<.001	232.18 (17.23)	<.001	286.08 (8.847)	<.001	302 (10.273)	.003
eGFR, mL/min/1.73m^2^	108.62 (13.62)	<.001	67.86 (5.5)	<.001	39.41 (9.32)	<.001	17.47 (3.12)	<.001	9.8 (2.67)	.002
Comorbidities										
HTN	65 (59.63%)		82 (75.22%)		92 (84.4%)		102 (93.57%)		104 (95.41%)	
IHD	22 (20.18%)		36 (33.02%)		64 (58.71%)		57 (52.29%)		48 (44.03%)	
CVA	14 (12.84%)		36 (33.02%)		14 (12.84%)		24 (22.01%)		37 (33.94%)	
PVD	3 (2.7%)		13 (11.92%)		11 (10.09%)		15 (13.76%)		22 (20.18%)	
Retinopathy	36 (33.02%)		48 (44.03%)		52 (47.7%)		79 (72.47%)		87 (79.81%)	
Neuropathy	14 (12.84%)		29 (26.6%)		48 (44.03%)		52 (47.7%)		63 (57.79%)	

Abbreviations: CKD, chronic kidney disease; CVA, cerebro-vascular accident; eGFR, estimated glomerular filtration rate; F, females; HbA1c, glycated hemoglobin; HTN, hypertension; IHD, ischemic heart disease; M, males; N, number; PVD, peripheral vascular disease; Q1-Q4, quartile 1-quartile 4; SD, standard deviation; T2DM, type 2 diabetes mellitus.

**Figure 2.**
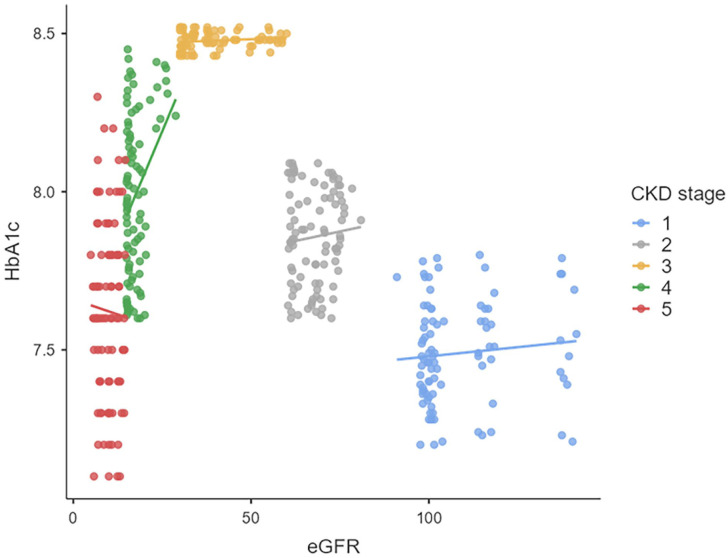
Scatterplots for correlation between eGFR and HbAic. Abbreviations: CKD, chronic kidney disease; eGFR, estimated glomerular filtration rate; HbAic, glycated hemoglobin.

Table 3 demonstrates the correlations between HbA1c and eGFR for all T2DM individuals with CKD stage 1 to 5. A weak positive correlation was identified between HbA1c and eGFR for patients in CKD stages 1 to 4 (*r* < .3). A negligible correlation was found between eGFR and HbA1c in CKD stage 5 (Figure 2).

**Table 3.** Correlation Between eGFR and HbA1c for T2DM Individuals With CKD Stage 1 to 5.

	HbA1c-CKD stage 1	HbA1c-CKD stage 2	HbA1c-CKD stage 3	HbA1c-CKD stage 4	HbA1c-CKD stage 5
Variables	(Spearman rho value)	(Spearman rho value)	(Spearman rho value)	(Spearman rho value)	(Spearman rho value)
eGFR	0.098	0.087	0.138	0.327	−0.036
*P* value	.307	.370	.042*	.001	.712

Abbreviation: CKD, chronic kidney disease; eGFR, estimated glomerular filtration rate; HbA1c, glycated hemoglobin; T2DM, type 2 diabetes mellitus.

* Marginal significance (unadjusted *P*-values between .01 and .05).

**Figure 3.**
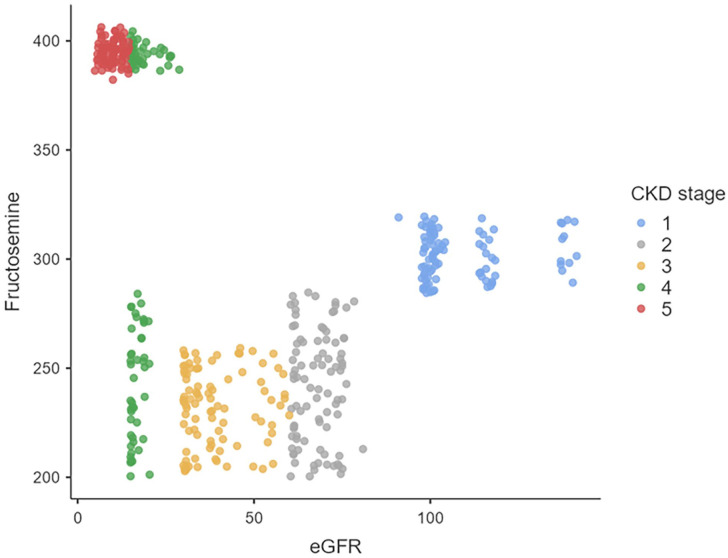
Scatterplots for correlation between eGFR and Fructosamine. Abbreviations: CKD, chronic kidney disease; eGFR, estimated glomerular filtration rate.

The correlations between eGFR and fructosamine for all T2DM patients with CKD stages 1 to 5 are shown in Table 4. A weak negative correlation was identified between eGFR and fructosamine in patients with CKD stages 1 to 5 (*r* < −.3). A weak negative correlation was found between fructosamine and eGFR (Figure 3).

**Table 4.** Correlation Between eGFR and Fructosamine for T2DM Individuals With CKD Stage 1 to 5.

	Fructosamine -CKD stage 1	Fructosamine -CKD stage 2	Fructosamine -CKD stage 3	Fructosamine -CKD stage 4	Fructosamine -CKD stage 5
Variables	(Spearman rho value)	(Spearman rho value)	(Spearman rho value)	(Spearman rho value)	(Spearman rho value)
eGFR	−0.041	−0.025	−0.392	−0.187	0.012
*P* value	.671	.793	.001^†^	.052*	.902

Abbreviations: CKD, chronic kidney disease; eGFR, estimated glomerular filtration rate; HbA1c, glycated hemoglobin; T2DM, type 2 diabetes mellitus.

† Bonferroni-adjusted α = .01.

* Marginal significance (unadjusted *P*-values between .01 and .05).

In the early stages of CKD (stages 1-2), the correlation between HbA1c and eGFR is weak to non-significant. As the disease progresses to stages 2 to 3, a weak to moderately negative correlation emerges, indicating that poor glycemic control may be associated with declining renal function. In advanced stages (4-5), the relationship becomes more complex due to the confounding factors such as anemia erythropoietin use and uremia.

Multivariate regression analysis was used to examine the relationship between significant variables and outcomes at various stages of CKD 1 to 5. Limited statistically significant associations were found in the results. Significantly, worse outcomes were linked to longer duration of diabetes in CKD 3 (β = −2.4, 95% CI [−4.7, −0.01], *P* = .04). HbA1c, fructosamine, age and hemoglobin were the only other variables that showed significant correlations across the phases of CKD with the majority of *P*-values surpassing 0.05 (Table 5).

**Table 5.** Multivariate Regression Analysis Between eGFR Across CKD Stages 1 to 5.

Variable	β estimate	95% CI	*P* value
CKD stage 1			
HbA1c	6.32	[-12.3, 24.3]	.5
Fructosamine	.06	[−0.2, 0.3]	.63
Age	.03	[−0.82, 0.89]	.93
Diabetes duration	1.57	[−2.0, 5.2]	.39
Hemoglobin	−.18	[−2.2, 1.8]	.85
CKD stage 2			
HbA1c	3.05	[−4.7, 10.8]	.43
Fructosamine	.01	[−0.03, 0.06]	.48
Age	.33	[−0.05, 0.7]	.08
Diabetes duration	−.07	[−1.2, 1.1]	.9
Hemoglobin	−.6	[−1.4, 0.1]	.1
CKD stage 3			
HbA1c	47.6	[−25.9, 121.2]	.2
Fructosamine	.02	[−0.08, 0.1]	.63
Age	−.09	[−0.69, 0.5]	.76
Diabetes duration	−2.4	[−4.7, −0.1]	.04
Hemoglobin	−.2	[-1, 0.6]	.63
CKD stage 4			
HbA1c	3.2	[−1.5, 8.1]	.18
Fructosamine	.001	[−0.01, 0.01]	.81
Age	−.02	[−0.18, 0.13]	.74
Diabetes duration	-0.12	[−1.2, 0.9]	.82
Hemoglobin	.06	[−0.25, 0.3]	.67
CKD stage 5			
HbA1c	−.48	[−2.5, 1.5]	.64
Fructosamine	.01	[−0.1, 0.1]	.8
Age	−.005	[−0.1, 0.1]	.95
Diabetes duration	.08	[−0.1, 0.2]	.38
Hemoglobin	−.05	[−0.2, 0.1]	.58

Abbreviations: β, multivariate regression estimate; CI, confidence interval; CKD, chronic kidney disease; HbA1c, glycated hemoglobin.

In CKD 2, hemoglobin displayed a non-significant trend (β = −.6, 95% CI [−1.4, 0.1], *P* = .1) while age showed a marginal trend toward significance (β = .33, 95% CI [−0.05, 0.7], *P* = .08). The confidence intervals for all other stages and variables contained the null value, indicating that there were no significant impacts. The broad confidence intervals for HbA1c in CKD 3 (95% CI [−25.9, 121.2] reveal significant uncertainty in the estimate for this stage (Table 5).

While other covariates did not exhibit consistent or significant relationships throughout CKD stages, the study indicated that the duration of diabetes may have an impact on CKD 3 outcomes. It might be necessary to conduct further studies with larger sample sizes to fully comprehend these relationships.

## Discussion

In this study, the accuracy of HbA1c and fructosamine as indicators of deteriorating renal function in the setting of T2DM was examined by comparing and assessing their associations with eGFR in individuals with CKD spanning stages 1 to 5. HbA1c and eGFR showed a weak positive correlation for patients in stages 1 through 4 of CKD. A weak negative correlation was seen between eGFR and HbA1c. There was a slight inverse relationship between eGFR and fructosamine in individuals with CKD stages 1 to 4. A weak positive correlation was found between fructosamine and eGFR.

The potential reasons for this correlation can be attributed to numerous reasons. HbA1c and eGFR are predicted to have little or no correlation in Stage 1 CKD, as eGFR usually ranges within normal or near-normal limits.^28^ This is due to the reason that there currently exists little impact of elevated HbA1c on eGFR. In this population, the correlation coefficient (*r*) between eGFR and HbA1c is likely to be weak to negative.^29^ This weak link can be attributed due to progressive nature of kidney damage brought on by hyperglycemia and compensatory mechanisms that keep eGFR within normal ranges even in the presence of continued damage.^30^ Maintaining appropriate glycemic management is essential to prevent progression to more advanced stages of CKD, where the impact on eGFR becomes more prominent, even though the correlation may be minimal in Stage 1.^30^ The correlation between fructosamine and eGFR is also anticipated to be weak, as fructosamine represents the short-term blood glucose levels over the previous 2 to 3 weeks.^31^ The relationship between fructosamine and eGFR is probably weak because kidney function is mainly conserved in Stage 1 and fructosamine levels are more controlled by blood glucose than renal function.

The effects of hyperglycemia on renal function are more noticeable in Stage 2 CKD, when eGFR is slightly decreased.^32^ Elevated HbA1c values are linked to more severe and progressive kidney impairment and are suggestive of inadequate glycemic management.^32^ Therefore, it is anticipated that HbA1c and eGFR will have a mildly to significantly negative correlation, with r values perhaps ranging from −.3 to −0.6.^33^ In contrast, our study’s correlation for CKD stage 2 individuals is just marginally positive. Poor glycemic management can cause eGFR readings to drop as renal function declines more quickly. The degree of albuminuria, age, comorbidities, duration of diabetes, medication use, and adherance are some of the variables that can affect the strength of the correlation.^32^ Likewise, it is anticipated that there will be a weak to moderately negative correlation between fructosamine and eGFR in Stage 2 CKD.^33^ The correlation values found in our study are consistent with these outcomes. As eGFR falls, fructosamine levels may rise somewhat due to decreased renal clearance of glycation products.^34^ However, the correlation may not be strong because eGFR is still mostly preserved.

The correlation between HbA1c and eGFR tends to be moderately negative as CKD advances to Stage 3, eGFR declines further, indicating moderate kidney dysfunction.^35^ This decline in kidney function is due to prolonged hyperglycemia and the development of diabetic nephropathy. In contrast, our study’s results showed a weak positive correlation (*r* = .138). Kidney damage can be made worse by poor glycemic control in T2DM, which can further reduce eGFR.^36^ Based on individual parameters including age, the duration of diabetes, and the existence of comorbidities like hypertension, the correlation’s strength can change. On the contrary, a weak to moderate correlation is anticipated for fructosamine and eGFR. Fructosamine levels may rise as a result of reduced fructosamine clearance resulting from declining renal function.^37^ The kidney’s capacity to eliminate fructosamine may be compromised as eGFR declines, which could lead to elevated fructosamine levels. Despite the existence of this association, non-glycemic variables impacting fructosamine levels and protein metabolism may still have an impact on the correlation.^37^ Our study found a negative correlation, which reflects the intricate interactions that exist between kidney function, glycemic management, and other non-glycemic variables.

eGFR is significantly decreased (15-29 mL/min/1.73 m^2^) in Stage 4 CKD.^38^ Similar to the results of our study, the correlation between HbA1c and eGFR frequently gets weaker or even insignificant. Because erythropoiesis-stimulating drugs, anemia, and altered hemoglobin metabolism can all impact HbA1c levels, the link between HbA1c and kidney function becomes more complicated.^39^ The actual blood glucose levels may be overestimated or underestimated as a result of these factors. The relationship may be further undermined by increased glycemic variability resulting from reduced insulin clearance in advanced CKD. In this stage, studies typically reveal weak negative correlations (*r* values ranging from −0.2 to −0.3).^40,41^ In Stage 4 CKD, fructosamine is likely to have an increasingly negative correlation (r values potentially ranging from −.3 to −.5) with eGFR.^42^ Our results suggest a weakly negative relationship between eGFR and fructosamine. Fructosamine levels may rise when renal function deteriorates because glycated protein removal is slowed down. However, because of factors like changed protein metabolism, variations in serum protein levels, and effects of fructosamine that are non-glycemic, the correlation might not be sufficiently strong.^43^ Advanced glycation end products (AGEs) contribute to renal damage in CKD by promoting oxidative stress and inflammation through their interaction with receptors like RAGE, which activates NADPH oxidase and NK-κB pathways, increasing reactive oxygen species (ROS), and pro-inflammatory cytokines. The accumulation of AGEs in CKD is due to their impaired clearance and heightened formation in hyperglycemic or uremic conditions. This leads to endothelial dysfunction, podocyte injury and fibrosis via TGF-β mediated extracellular matrix deposition, accelerating kidney function decline. Antioxidants, such as grape seed extract, have shown potential in mitigating AGE-induced damage.

Ultimately, when eGFR falls below 15 mL/min/1.73 m^2^ in Stage 5 CKD, the correlation between HbA1c and eGFR is expected to be either significantly negative or almost nonexistent.^44^ These results align with the comparable outcomes seen in our study. The interpretation of HbA1c values is made more difficult at this point due to the kidneys’ substantially decreased capacity to filter and eliminate toxins.^45^ HbA1c measurements can be impacted by uremia, specific drugs, and other conditions, which reduces their validity as markers of glycemic management.^45^ In early stages of CKD, there may have been a moderate to weak negative correlation; however, in Stage 5, this relationship becomes less consistent. Research findings indicate r values ranging from −.2 to −.4^46^; however, these can differ considerably according on patient characteristics and study design. The relationship between fructosamine and eGFR in Stage 5 CKD is expected to be moderately negative. According to our study, the r values are as near to very weak correlation as .012 in our population. At this stage, fructosamine levels may be indicative of impaired protein metabolism, hypoalbuminemia, which is frequent in severe CKD, as well as poor glycemic management. Because of decreased glycated protein clearance, lower eGFR may be linked to increased fructosamine levels; however, the relationship is regulated by more than just renal function and glycemic management.^46^

Fructosamine has several benefits for diabetic care monitoring, particularly for CKD patients with stages 1 through 5.^47^ It is a useful marker for analyzing recent changes in glycemic control, which is essential for rapidly monitoring the effects of medication modifications. It reflects average blood glucose levels over the past 2 to 3 weeks.^48^ Fructosamine is a more accurate test in patients with hemoglobin variations, anemia, or other disorders affecting red blood cell turnover than HbA1c is. These conditions are common in advanced CKD.^49^ Furthermore, fructosamine is particularly useful in specific medical situations when HbA1c is less reliable, such as in patients with severe blood loss, recent blood transfusions, or during pregnancy.^49^ Emerging research also suggests that fructosamine levels may predict CKD progression, with elevated levels associated with more rapid reductions in renal function.^50^ Fructosamine testing is a useful tool for monitoring individuals with changing glucose levels since it is also sensitive to acute glycemic fluctuation and is affordable, frequently less expensive than HbA1c.^51^ Its capacity to react rapidly to diabetes treatments enables prompt therapeutic modifications, thereby lowering the risk of complications that are brought on by poor glycemic control.^51^

In light of our discussion, HbA1c appears to be a more clinically significant predictor than fructosamine of a CKD patient’s decline in type 2 diabetes mellitus during stages 1 through 5. This is primarily because HbA1c can accurately indicate long-term glycemic management. HbA1c is a comprehensive measure for controlling chronic hyperglycemia because it provides a summary of levels of blood glucose over the preceding 2 to 3 months. Chronic hyperglycemia is a significant risk for CKD and the HbA1c test quantifies the long-term impacts of poor glucose control on renal function.^42^

In the early stages of CKD (stages 1-2), the correlation between HbA1c and eGFR is weak to non-significant. As the disease progresses to stages 2 to 3, a weak to moderately negative correlation emerges, indicating that poor glycemic control may be associated with declining renal function. In advanced stages (4-5), the relationship becomes more complex due to confounding factors such as anemia erythropoietin use and uremia. Even while anemia and altered hemoglobin metabolism cause the correlation between HbA1c and eGFR to diminish in advanced stages of CKD stages 4 to 5, HbA1c is still important for determining the risk of additional renal impairment. Our findings highlight the need for cautious clinical evaluation, although the test’s overall ability to predict the course of the disease is not negatively impacted by these challenges in interpreting HbA1c in late-stage CKD.

However, because fructosamine only represents short-term glycemic management over a period of 2 to 3 weeks, its capacity to predict long-term trends in kidney function is restricted.^52^ Even though fructosamine may partially correlate with eGFR, especially as CKD progresses and renal clearance of glycation products decreases, fructosamine has a lower predictive value than HbA1c.

In clinical practice, HbA1c is widely used to monitor the management of diabetes and associated comorbidities, particularly CKD. Its extended time horizon and well-established function make it a more reliable indicator for predicting the way individuals with type 2 diabetes would manage the advancement of their CKD.

Our study provides a comprehensive relationship between fructosamine, eGFR, and HbA1c in individuals at various stages of CKD with T2DM. Our research also contributes significantly to the significance of the long-term impact of glycemic control on renal function. The findings are more likely to be credible since they are derived from a large sample of CKD patients of all stages. Our study’s specificity and depth are further enhanced since we differentiated different stages of CKD and their corresponding relationship with fructosamine and HbA1c. In advanced CKD, clinicians ought to consider using fructosamine as an adjunct to supplemental HbA1c, especially when hemoglobin abnormalities or rapid treatment modifications are required.

Our study has several limitations, despite its numerous benefits. One of the potential constraints that we identified is the likelihood of confounding variables impacting the associations between fructosamine, eGFR, and HbA1c. The management of T2DM in CKD depends on age, diabetic duration, other concomitant illnesses including hypertension and pharmacotherapy. The blood creatinine levels and dehydration in our study may cause the eGFR readings to fluctuate. Furthermore, it is challenging to establish a causal link between glucose regulation and renal function due to the cross-sectional study design. Focusing solely on fructosamine and HbA1c as substitutes for glycemic control, without taking into consideration additional indicators (like glycated albumin), could limit the thoroughness of the glycemic evaluation Fructosamine levels may be influenced by hypoalbuminemia in advanced CKD; future studies should include serum albumin measurements to adjust for protein status. Cause-and-effect interfaces are not possible because of the cross-sectional design. Since advanced CKD patients (stage 4-5) are more likely to seek treatment, selection bias may occur, thereby overrepresenting symptomatic cases. It is not possible to rule out reverse causality, such as the severity of CKD impacting glycemic indicators. In order to confirm these relationships, longitudinal research is required.

While we applied Bonferroni correction to primary analyses, some secondary comparisons remain susceptible to Type I errors due to multiple testing. Unadjusted *P*-values between .01 and .05 should be interpreted cautiously, particularly for exploratory relationships. Future studies with larger samples could employ more powerful corrections (eg, false discovery rate control). Another limitation is the lack of adjustment for serum albumin levels, which may influence fructosamine measurements, particularly in advanced CKD. Future studies should account for protein status to refine the reliability of fructosamine as a glycemic marker in renal impairment.

Future research should focus on conducting longitudinal studies to investigate the correlation between renal function and glycemic control in CKD with T2DM. Studies venturing into the roles of other biomarkers such as cystatin C, glycated albumin, and advanced glycation end-products, could provide a more comprehensive understanding of the correlation between glycemia and renal dysfunction. A deeper insight regarding the way various interventions affect CKD progression among individuals with T2DM could be obtained by understanding the impact of specific diabetic medications and other confounding variables. Assessing the influence of non-glycemic factors on fructosamine levels and their correlation with renal function could improve the predictive value of fructosamine as a biomarker in clinical settings.

The stage of kidney disease must be taken into consideration by clinicians while monitoring glycemic control in T2DM patients with CKD. While fructosamine can assist in capturing recent changes in blood sugar, such as following medication adjustments, HbA1c is still the gold standard for evaluating long-term glucose management in early CKD (stages 1-2). HbA1c becomes less reliable as kidney function deteriorates to stages 3 to 4, as its results may be influenced by anemia, erythropoietin medication, or other CKD-related variables. Fructosamine is an invaluable asset in this situation, but if the patient has low albumin levels, its values should be interpreted with caution. By stage 5 CKD, HbA1c generally loses all dependability due to uremia or blood cell abnormalities. Continuous glucose monitors (CGMs) or glycated albumin (when available) may be used to bridge the gap in these circumstances. Fructosamine still plays a role, but it must be evaluated in the context of the patient’s overall protein status and clinical picture. Finally, a flexible strategy of focusing on HbA1c early, combining markers mid-disease, and switching to alternatives later can strike a compromise between accuracy and the realities of CKD consequences.

## Conclusion

The study provides an insight into the significance of HbA1c and fructosamine as a clinically effective marker for tracking the progression of CKD in individuals with T2DM. Initially, we identified a weak positive correlation between HbA1c and eGFR in the early stages of CKD. However, the correlation strengthened along with CKD progression. This makes it imperative to underscore the critical need to maintain optimal glycemic control to halt the progression of renal impairment. While fructosamine offers insights into short-term glycemic control, its predictive value for long-term kidney function is lower than that of HbA1c. The findings obtained in our study, highlight the importance of interpreting glycemic indicators with utmost caution, especially in the advanced stages of CKD. This is because factors like hemoglobin metabolism abnormalities and anemia can influence HbA1c levels. The weak negative correlation (*r* = −.036) in CKD stage 5 indicated that uremia, erythropoietin usage and anemia all impair HbA1c reliability. Although it is still impacted by hypoalbuminemia, fructosamine may provide complementary short-term insights. In addition, we also identified that fructosamine can serve as a supplementary marker with limited predictive ability. HbA1c and fructosamine are essential tool for monitoring diabetes care and predicting the trajectory of CKD.

## Supplemental Material

sj-docx-1-end-10.1177_11795514251362516 – Supplemental material for Relationship Between Glycemic Indices and eGFR Values Among Type 2 Diabetes Mellitus Individuals With Chronic Kidney Disease Across Various Progression Stages Supplemental material, sj-docx-1-end-10.1177_11795514251362516 for Relationship Between Glycemic Indices and eGFR Values Among Type 2 Diabetes Mellitus Individuals With Chronic Kidney Disease Across Various Progression Stages by K. Vaishali, Chandana Acharya, Shobha U. Kamath, Revati Amin and Shivashankara Kaniyoor Nagri in Clinical Medicine Insights: Endocrinology and Diabetes
